# A case report of thumb weakness misdiagnosed as radial neuroma and resulting in upper limb dysfunction after surgery

**DOI:** 10.3389/fresc.2026.1747273

**Published:** 2026-03-30

**Authors:** Zhiqi Lin, Keyun Gan, Yuanbin Yang, Longyue Yi, Wei Yao

**Affiliations:** 1 First Department of Rehabilitation, Wangjing Hospital, China Academy of Chinese Medical Sciences, Beijing, China; 2 School of Sports Medicine and Rehabilitation, Beijing Sport University, Beijing, China

**Keywords:** case report, dysfunction, electroacupuncture, radial neuropathy, ultrasonography

## Abstract

A 37-year-old female patient was misdiagnosed with radial neuroma due to thumb weakness. After surgery, the weakness of her upper limbs worsened over 4 months. The most obvious symptoms upon admission were drooping wrists and inability to straighten the five fingers. Electromyography showed left radial nerve injury. She was preliminarily diagnosed with radial neuritis. The patient was treated with ultrasound-assisted electroacupuncture for 6 months, and the muscle strength of the upper limbs increased significantly. This case suggests that ultrasound-guided localization and electroacupuncture therapy are the key methods in treating radial neuritis.

## 1 Introduction

The patient was misdiagnosed with radial neuroma and underwent surgery due to difficulty in lifting the thumb. Postoperatively, she presented with wrist extension and finger extension impairments. After ineffective physical and manual therapy, she was admitted to our hospital, where she received ultrasound-guided electroacupuncture for 6 months, and her upper limb muscle strength improved significantly. This case is unique due to an unknown etiology and the unresponsiveness of the patient's postoperative neuritis to conventional rehabilitation. Furthermore, most current reports focus on electroacupuncture for the brachial plexus, with relatively few reports on the radial nerve. A detailed report follows.

This case report was prepared in accordance with the CARE Guidelines (13-item checklist), which provide a standardized framework for developing case reports (1).

## 2 Case narrative

In December 2024, a 37-year-old female patient engaged in nursing work suddenly developed left thumb flexion and extension disorders and was treated at another hospital. Intraoperatively, no radial neuroma was found; instead, nerve adhesions were observed, and a radial nerve release procedure was performed. Radial neuritis was suspected postoperatively. She presented with thumb numbness, limited extension of the five fingers, preserved flexion function, varying degrees of upper limb muscle weakness, and prominent wrist drop with inability to extend the five fingers. Physical and manual therapy failed to alleviate her symptoms. She was admitted to this hospital on January 22, 2025, and at admission, she had thumb numbness, impaired thumb function, impaired finger extension, limited wrist elevation, and limited shoulder abduction.

After physical examination, it was found that the patient's left radial nerve distribution area had decreased pinprick sensation, and a transarticular scar about 15 cm long was found in the left elbow joint. Palpation revealed a firm texture of the scar and atrophy of the left hand muscles. Further manual muscle testing of the left upper limb showed that the strength of the left wrist extension, finger extension, and thumb extension were weakened. Brachioradialis hyporeflexia, positive radial nerve tone test, negative Tinel sign. The Radial Nerve Function Score (RNFS) was 37. In particular, the scores of muscle strength and daily function were significantly decreased, which were 8 points (out of 40) and 5 points (out of 30), respectively.

In addition, in order to further confirm the cause of neuritis in the patient, the patient underwent blood tests (including C-reactive protein, complement C2, three immunoglobulin (Ig) items, and antinuclear antibody spectrum.), No obvious abnormalities were found on her magnetic resonance imaging (MRI) and electrocardiogram (ECG).

## 3 Intervention modalities

### 3.1 Ultrasound-guided electroacupuncture intervention

**Figure 1:**
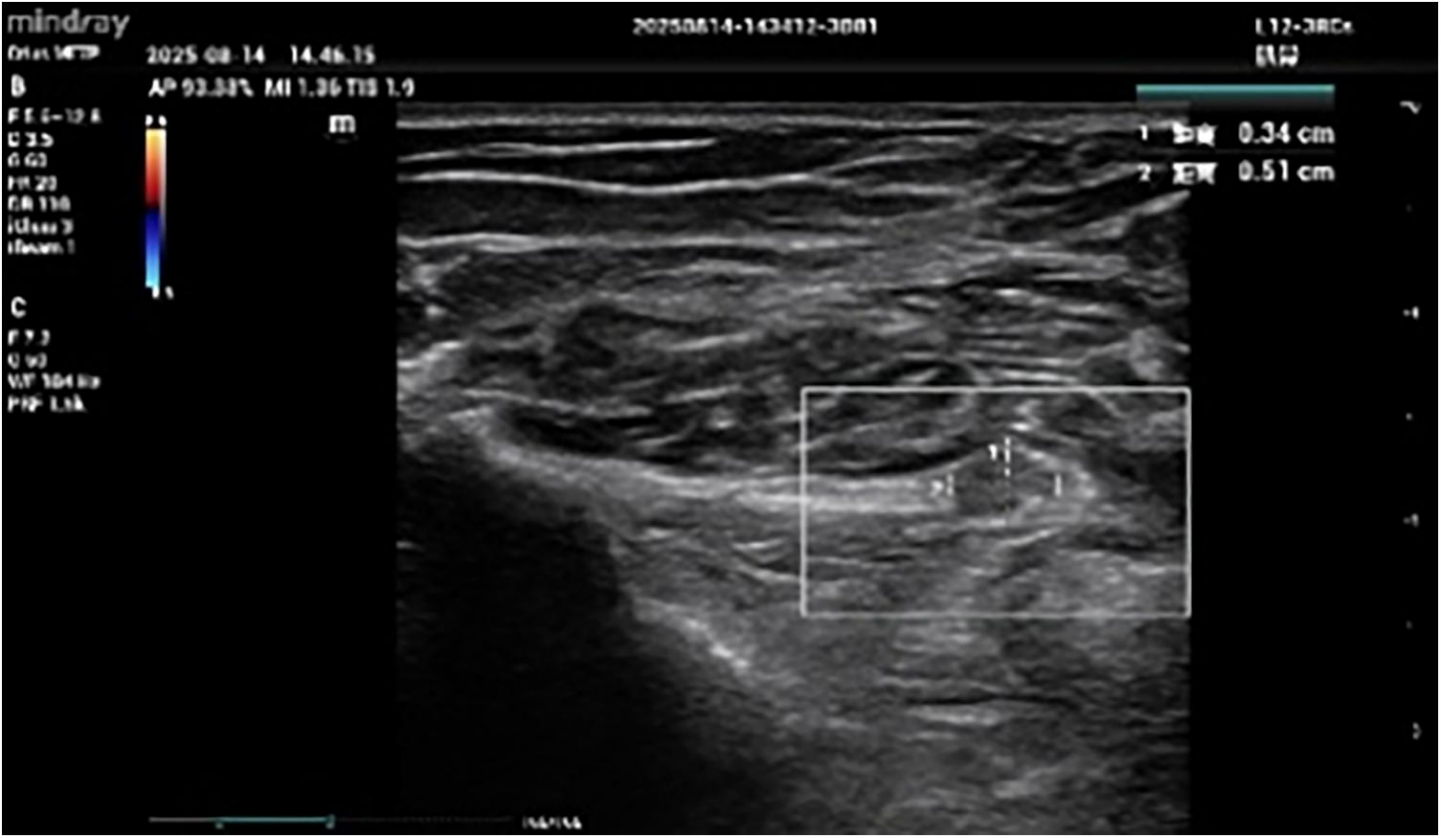
Ultrasound shows the abnormal location of the radial nerve. This figure displays the site of radial nerve thickening in the patient identified via ultrasound, which is one of the radial nerve thickening sites marked in the legend. All subsequent acupuncture treatments for the patient were adjusted based on the thickened nerve sites detected by ultrasound.

The patient was treated with stainless steel acupuncture needles (size: 0.3 mm × 40 mm) inserted to a depth of approximately 0.5 cm. Based on the *Huangdi Neijing·Suwen* (Yellow Emperor's Internal Classic·Plain Questions) and its *Atrophy Treatise*, which emphasizes that “treating atrophy requires focusing on the Yangming Meridian”, the main acupoints selected were from the Large Intestine Meridian of Hand-Yangming (including Jianyu, Quchi, and Shouwuli etc.) (Table 1). At the same time, acupuncture was performed on the thickened part of the radial nerve shown by ultrasound and the part with unclear boundaries with surrounding tissues during the course of the radial nerve through ultrasound positioning (Figure 1). The electroacupuncture instrument was initially set to zero, and the continuous wave mode was selected with a frequency of 5 Hz and a current of 0.3–0.8 mA. The intensity was gradually increased based on the occurrence of local rhythmic muscle contractions until the patient could tolerate finger extension movements. Each session lasted 25 min, with treatments administered every 2 days for 5 months (Table 2).

**Table 1:** Details of acupuncture points.

Acupoint	Positioning
Jianyu (LI15)	Depressions in the shoulder joints
Shouwuli (LI13)	On the outside of the arm, On the line of Qu Chi and Jianyu
Quchi (LI11)	At the lateral end of the cubital crease, it is at the midpoint of the line between the Chize point and the lateral epicondyle of the humerus when the elbow is flexed
Shousanli (LI10)	On the radial side of the back of the forearm, On the line between Yangxi and Quchi
Waiguan (SJ5)	The horizontal lines on the back of the wrist cross striations are two inches high
Hegu (LI4)	On the outside of the back of the hand, the second metacarpal is near the midpoint on the side of the thumb

**Table 2:** Timeline.

Category	Time	Key events
Surgery-related	December 2024	Sudden thumb flexion and extension impairment occurred; initial consideration of radial neuroma, followed by surgery.
		No radial neuroma found intraoperatively; nerve decompression was performed due to suspected nerve adhesion caused by radial neuritis.
	Post-surgery (December 2024)	Symptoms worsened: varying degrees of muscle strength loss in the upper limb, with wrist drop and inability to grasp with five fingers being the most prominent.
Rehabilitation training	December 2024–January 2025	Received rehabilitation training; no obvious improvement in muscle strength or limb function was observed.
Electroacupuncture & TMS	January 2025	Received electroacupuncture therapy at this hospital.
	February 10–March 5, 2025	Underwent 15 sessions of transcranial magnetic stimulation (TMS); significant improvement in muscle strength was observed after the treatment.
	June 2025	Completed the entire course of electroacupuncture and TMS treatment.
Follow-up	August 2025	Follow-up: The patient reported returning to work; all functions recovered normally except for difficulty in performing fine movements that require the thumb.

### 3.2 Other interventions

Concurrently with electroacupuncture, ultrasound (0.5–1.0 MHz, 2–3 sessions per week, 15 min per session) was applied for 4 months to promote nerve regeneration and scar release. To improve the peripheral nervous system, 15 magnetic stimulations were conducted over 1 month, targeting the central and peripheral regions such as the scalp, brachial plexus and superficial radial nerve (C7, Erb's point, elbow, abductor pollicis brevis muscle).

## 4 Post-treatment outcomes

After 6 months of treatment, the patient's muscle strength was restored, and the recovery was more obvious with wrist extension and four-finger extension (Table 3). The brachioradial reflex was more brisk than before treatment, the radial nerve tone test was positive, but the induced pain was reduced, and the Tinel test was negative. Ultrasound showed no significant change in the thickness of the left radial nerve (0.13–0.42 cm). Electromyography showed an increase in the sensory transmission velocity of the left radial nerve (57.0 m/s before surgery, 54.1 m/s after surgery, 54.1 m/s 3 months after electroacupuncture intervention, 55.5 m/s after electroacupuncture intervention), and an increase in amplitude (28.9 μV before surgery, 10.3 μV after surgery, 10.9 μV 3 months after electroacupuncture intervention. 12.4 μV 5 months after electroacupuncture intervention). The recovery of sensory transmission mainly occured between 3 and 6 months after the intervention. Meanwhile, the electromyography results showed that compared with before treatment, the amplitude of the distal compound muscle action potential (CMAP) of the left radial nerve increased. The patient has returned to work and is able to complete occupation-related work independently, only using the right finger to assist in the completion of the left thumb extension movement every time it is necessary to complete the movement. The Radial Nerve Function Score (RNFS) was 77 (Table 4). Partial recovery of muscle strength and daily function was more obvious. No adverse events were observed during the treatment period.

**Table 3:** Comparison of muscle strength before and after treatment.

Muscle group	Muscle strength test before intervention	Muscle strength test after intervention
Shoulder abduction	4−	4
Forearm supination	4	5
Wrist joint extension	2−	4
Four fingers extended	1+	4
Thumb out	1	1+

**Table 4:** Comparison of the radial nerve function scale scores before and after treatment.

Scoring section	Motor function	Sensory function	Daily life	Total
Before	8	24	5	37
After	27	30	20	77

## 5 Discussion

Before the operation, the patient experienced symptoms such as weakness in the thumb and decreased sensation. It was considered that it might be stroke, radial neuritis, radial neuroma, or cervical spondylosis of the nerve root type (C6 nerve root involvement). Cervical spondylosis of the nerve root type is often accompanied by radiating pain in the neck, and the neck imaging shows normal. Therefore, this disease was not considered for this patient. At the same time, a cranial imaging examination was conducted on the patient, ruling out the possibility of central nervous system diseases.

When the patient first received treatment, tumor-like images of the radial nerve were shown, suggesting a radial neuroma. Intraoperatively, the tumor-like thickening at the radial nerve site was found to be secondary to inflammation, rather than a true radial neuroma. Therefore, a nerve release surgery was performed to relieve nerve compression. Although both radial neuritis and radial neuroma may present with pain, paresthesia, or motor dysfunction in the distribution of the radial nerve, they are distinct clinicopathological entities that require careful differentiation. Peripheral neuritis refers to peripheral inflammatory and degenerative diseases caused by various pathologies. The radial nerve (RN) causes “wrist drop” when injured this manifests as weakness of the extensor carpi radialis longus and brevis muscles. In contrast, radial neuroma refers to a benign neoplastic or reactive lesion arising from the radial nerve, usually manifesting as a slow-growing, localized mass with relatively mild or absent inflammatory signs. Nerve dysfunction (sensory and motor dysfunction) is often gradual and related to local compression by the tumor. Histopathology remains the gold standard for differentiating the two diseases. Based on the experience from this case, if peripheral nerve imaging reveals tumor-like hyperdense lesions and clinical progression of neurological dysfunction cannot be determined, histopathological examination should be prioritized to differentiate between a neuroma and tumor-like thickening secondary to neuritis.

There are four main causes of peripheral neuritis. Diabetes is the most common cause of peripheral neuritis in Europe and North America. The prevalence of alcohol-associated polyneuropathy in patients with chronic alcoholism is 22%–66%. Chemotherapy-induced neuropathy (CIN) is also becoming more common clinically with a prevalence of 30%–40% due to the increasing prevalence of malignant diseases and the use of new chemotherapy drugs, which varies widely depending on the drugs and treatment regimens used. Peripheral neuritis can also be caused by genetic causes or as a result of vitamin deficiency or excess, exposure to toxic substances and certain medications, and various immune processes (2). During this period, the patient in the study underwent C-reactive protein and antinuclear antibody profile etc., to ruled out immune system causes. MRI examination excluded lesions and compression of soft tissues. An ECG test ruled out symptoms caused by heart disease. Although the symptoms worsened after nerve release surgery, the original cause of the inflammation has not yet been found.

The nervous system remodeling-related mechanisms of acupuncture in the treatment of peripheral nerve injuries (PNI) are mainly divided into two aspects: the regeneration and repair of peripheral nerve fibers and the spinal cord regulation mechanism in PNI (3).

Neuronal networks have evolved to control functions. One is called electroacupuncture (EA) the technique in which specific acupuncture points on the body are electrically stimulated has long been used to activate these networks that regulate the function of certain organs to treat various diseases. Many studies have shown that EA can alleviate neuroinflammation both central (Alzheimer's disease, spinal cord injury, Parkinson's disease, and vascular dementia) and peripheral (e.g., after surgical injury or lipopolysaccharide injection). Electroacupuncture involves the treatment of about 13 types of peripheral neuropathy, with facial paralysis and facial spasm being the most common, followed by trigeminal neuralgia and sciatica, almost all 13 diseases have an effective rate of more than 90% (4). Studies have shown that electroacupuncture can effectively promote the regeneration and repair of injured peripheral nerves, and 5 Hz electroacupuncture has the best effect. Its mechanism may be related to the recovery of axoplasmic flow after sciatic nerve transection in rats, the proliferation of Schwann cells, the growth of nerve fibers and many other pathways. In the waveform of electroacupuncture, continuous wave and disperse-dense wave were mainly used. Compared with disperse-dense wave, low-frequency continuous wave has a more significant effect on the regeneration and functional recovery of PNI. For the study of current intensity, there are few relevant literature, and the appropriate intensity is to achieve slight muscle contraction (5). Taking into account a series of factors such as the current tolerance of the patient in this study comprehensively, the patients in this study were treated with 5 Hz continuous waves and currents ranging from 0.3 to 0.9 mA.

Two cases related to the use of electroacupuncture in the treatment of movement disorders caused by peripheral neuritis were reported in PubMed (6, 7), both of which achieved good therapeutic effects. In particular, the treatment of radial neuritis is relatively rare, and the two retrieved articles are cases of brachial plexus nerve injury. Chao Wang et al. and Po-Hsuan Su et al. reported a 46-year-old female with left shoulder strain complicated by peripheral nerve inflammation and a 26-year-old male with pathologically confirmed brachial plexus neuritis following tetanus, diphtheria, and pertussis (TDP) vaccination, respectively. Both cases were treated with electroacupuncture under ultrasound guidance. After the intervention, pain, muscle strength and activities of daily living (ADLs) were all improved. The cases of peripheral neuritis treated with electroacupuncture mentioned above were reviewed and the actual clinical conditions were analyzed. In this study, the patient received electroacupuncture treatment under ultrasound guidance.

Repetitive transcranial magnetic stimulation (rTMS) can stimulate damaged nerve tissue, promote axon regeneration, and accelerate the recovery of nerve function. Therefore, magnetic stimulation was adopted to treat the patient in this study. According to neuroplasticity, it contributes to sensorimotor recovery after peripheral nerve damage (PNI) (8). Mohanty et al. proposed that in the case of input loss due to peripheral injury, plasticity consists of two main components. The first part is the reduction of neuronal activity in the lesion projection area, and the second part is the reduction of neuronal activity, which allows the damaged area to respond to new stimuli through axonal growth and synaptic strengthening mechanisms. The achieved input remapping makes the stimulated area of the damaged area similar to that of the cells within the spatially adjacent undamaged area in the cerebral cortex (9). Peripheral mixed nerve stimulation may cause combined activity of somatosensory afferents and intrinsic motor cortical circuits. This combination appears to be particularly effective in modulating motor output (10). To promote the peripheral nerve plasticity of the patient in this study, 15 central and peripheral magnetic stimulations were performed within 1 month.

This single-patient treatment case has limitations: The patient received a series of treatments including electroacupuncture and rTMS simultaneously. It is impossible to determine whether it is the result of a single treatment, and it is even possible that it is the patient's natural recovery within 6 months. It cannot rule out accidental improvement in efficacy, with one-sided effectiveness evidence. Ultrasound guidance during treatment is handled by different devices at different times, which has differences.

## 6 Conclusion

In this case, ultrasound-assisted electroacupuncture achieved a remarkable therapeutic effect in the treatment of postoperative radial neuritis. However, due to the single-case design, spontaneous recovery and individual variability cannot be excluded. Thus, ultrasound-guided electroacupuncture should be individualized based on the specific clinical condition of each patient with radial neuritis.

## Data Availability

The original contributions presented in the study are included in the article/Supplementary Material, further inquiries can be directed to the corresponding author.
